# Differential fall migratory routes of adult and juvenile Ipswich Sparrows (*Passerculus sandwichensis princeps*)

**DOI:** 10.1186/s40462-016-0067-8

**Published:** 2016-01-21

**Authors:** Zoe J. Crysler, Robert A. Ronconi, Philip D. Taylor

**Affiliations:** Department of Biology, Acadia University, Wolfville, B4P 2R6, NS Canada; Canadian Wildlife Service, Environmental Stewardship Branch, Environment Canada, 45 Alderney Drive, Dartmouth, B2y 2N6, NS Canada; Bird Studies Canada, Port Rowan, N0E 1M0, ON Canada

**Keywords:** Migration, Age, Route, Automated telemetry, Songbird, Stopover, Ipswich Sparrow

## Abstract

**Background:**

Island breeding birds present an ideal system for studying migratory movements in passerines because their populations are clearly demarcated, and individuals must depart on migration from a single location. The Ipswich Sparrow (*Paserculus sandwichensis princeps*) is a subspecies of the Savannah Sparrow that breeds exclusively on Sable Island, Nova Scotia, Canada and winters along the Atlantic coast of North America. We used a network of 34 automated VHF telemetry receivers to track radio-tagged adult and juvenile Ipswich Sparrows from their breeding island southward through the first half of their fall migratory journey.

**Results:**

We compared adult to juvenile timing and routes. We show that juveniles leave the island approximately 24 days prior to adults and remain temporally separated from them during migration through Nova Scotia. Juveniles have different overwater orientations that result in migratory routes with shorter ocean crossings and a longer overall distance travelled compared to adults. Juveniles also have more frequent and longer stopovers, and displayed some reverse migration.

**Conclusion:**

We demonstrate that migratory routes differ between adults and juveniles, suggesting that routes change as individuals age, possibly through learning or social interactions. These differential routes also suggest that sparrows experience risk in different ways with juveniles selecting shorter overwater flights with less navigational risk at the cost of increased time spent in migration.

**Electronic supplementary material:**

The online version of this article (doi:10.1186/s40462-016-0067-8) contains supplementary material, which is available to authorized users.

## Background

In passerine birds, it has been suggested that 85 % of annual mortality can occur during the migration season [1, 2] which suggests that there should be strong selection for adaptations to reduce mortality during that period. Selection could act on the timing of migratory flights, the choice of stopover sites, and the choice of migratory routes. Under optimal migration theory, these decisions are based on trade-offs between the need to accumulate energy at high-quality stopover sites, to avoid predation, and to select for good weather conditions [3, 4]. What constitutes an optimal migratory route appears to vary between species. For example, some passerines appear to minimize time costs [5], while others minimize energetic costs [6].

Adult and juvenile songbirds have different experience and abilities [7, 8]. It can therefore be expected that their perception of the relative risks and benefits of options during migration may also differ. Compared to adults, juveniles may be less able to avoid predators [9] and orientate in appropriate directions [10], have more variability in their orientation [11], and take longer and more frequent stopovers [12, 13]. In addition, both adults and juveniles are known to undertake flights in inappropriate migratory directions in both spring and fall [14–16]. Such ‘reverse migrations’ and ‘landscape-scale stopover movements’ may be due to orientation errors, overshooting targets, or for gathering information about the environment [17, 18].

There is considerable support for the hypothesis that during migration, juvenile migratory songbirds orient using magnetic and celestial cues [19–21]. Translocation experiments suggest that it is primarily adults who can re-orient en route, possibly due to a learned navigational map based on magnetic cues [10, 22]. Thus, as they age and gain experience, individuals can correct ‘mistakes’ in their initial migrations by altering routes that optimize trade-offs—for example, avoiding barriers, more rapidly locating suitable stopover sites, and taking better advantage of weather to facilitate movement.

Behavioural differences between adults and juveniles may also be magnified when ecological barriers such as oceans are encountered [23, 24]. Long overwater flights present a challenge to migratory songbirds since they must acquire the necessary energy reserves to complete a non-stop flight as well as orient correctly [15, 23, 25]. Thus, small differences in adult and juvenile ability and experience could result in large observable differences in behaviour when open water is encountered en route. Altered behaviour at open water could include longer stopovers, differential route choice [26], or movements to locations with more food and fewer predators [14, 25, 27]. Because they have experience, it thus follows that adults may display less variability in orientation, fewer reverse migration or landscape-scale stopover movements en route, and less time at stopovers at ecological barriers.

**Fig. 1:**
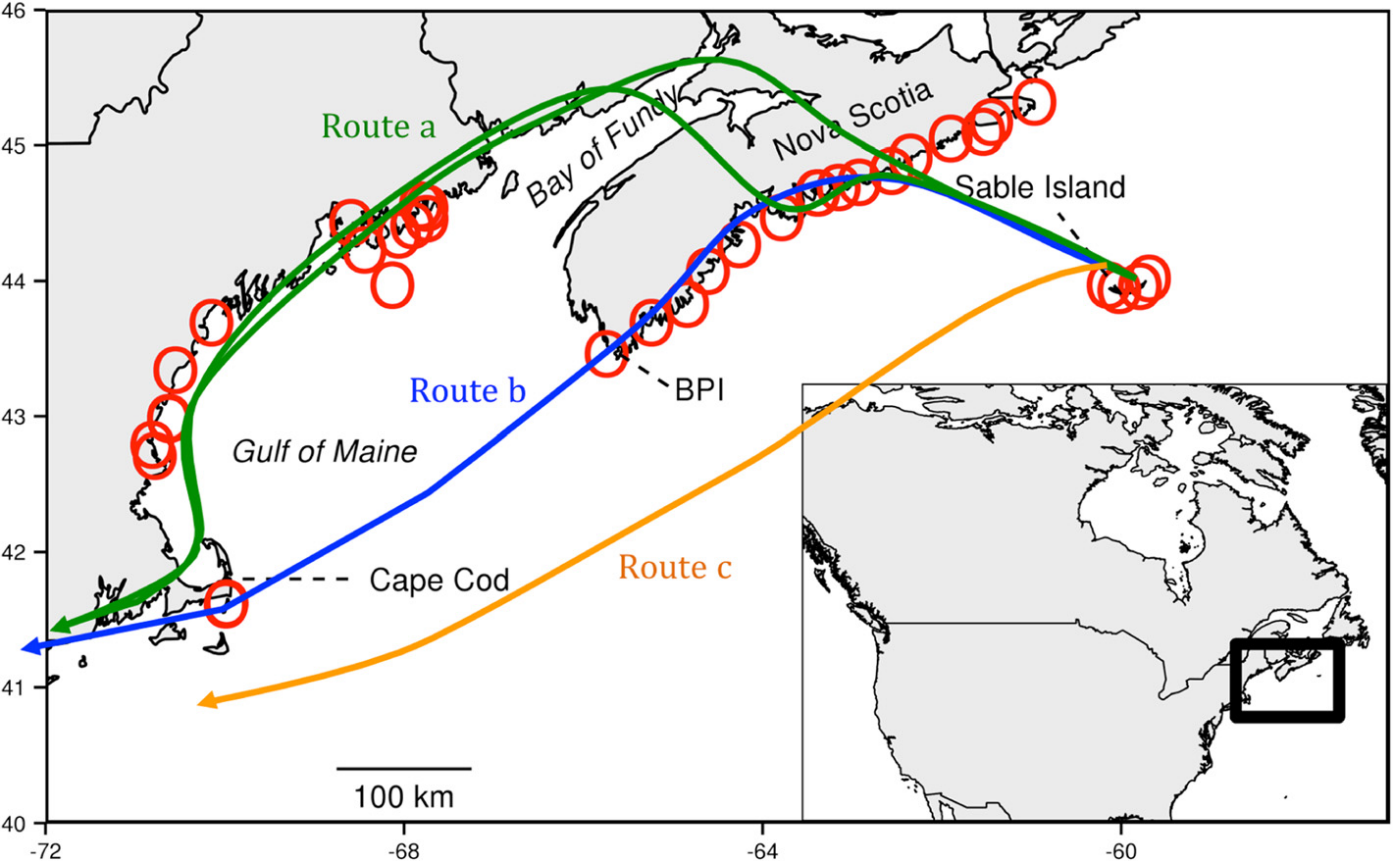
Map of telemetry array and potential migratory routes of Ipswich Sparrows. Potential migratory routes of Ipswich Sparrows including route a; land-based (*green line*), route b; coastal across the Gulf of Maine (*blue line*), and route c; direct to Cape Cod (*orange line*). *Red circles* represent receiver sites. Bon Portage Island = BPI

We studied an endemic island passerine that undergoes a yearly migration typical of most short-distance temperate migrants. The Ipswich Sparrow (*Passerculus sandwichensis princeps*) is a subspecies of Savannah Sparrow that breeds exclusively on Sable Island, Nova Scotia, Canada and winters in coastal dunes along the eastern seaboard of North America [28, 29]. Ipswich Sparrows migrating south in the fall must cross or avoid two large water bodies (Fig. 1). Their options are to fly northwest to the Nova Scotia mainland and travel directly west to either cross or circumvent the Bay of Fundy (route a), travel south along the southwest coast of Nova Scotia to cross the Gulf of Maine (route b), or migrate directly overwater from Sable Island to Cape Cod, MA (route c). Observations during fall migration (www.ebird.ca) indicate that at least some individuals are detected along the Bay of Fundy suggesting partially overland routes through Nova Scotia. Which individuals select these routes is unknown, but we propose that age may help explain the observed differential routes because of the aforementioned differences in relative risks and benefits of overwater flights.

Using automated digital telemetry [25] we compared the timing of initiation of migration, the routes chosen, and the behavioural decisions en route (stopover behaviours, decisions at two major water crossings, extent of ‘reverse’ migration) of adult and juvenile Ipswich Sparrows. The differences allow us to infer to what extent passerines learn and adjust their routes as they age.

## Methods

### Study sites and methods

Sable Island (44°N, 60° W) is situated off the coast of Nova Scotia, approximately 160 km from the nearest mainland, in the Northern Atlantic Ocean. It is a roughly 42 km long crescent shaped sandbar that is 1.5 km across at its widest point. Just under half the surface is covered in vegetation comprising shrubs, forbs, and grasses [28].

We radio tagged 64 Ipswich Sparrows (16 adult male, 15 adult female, 33 fledglings) between 12–18 August 2013 on Sable Island. To improve the odds that tracked juveniles would survive until the onset of migration [30] only early brood juveniles (those likely from the first or second broods) were tagged.

Ipswich Sparrows winter along Atlantic coast of North America with the highest densities being found between New Jersey and Virginia. They migrate north to arrive on Sable Island in late April and begin fall migration in late September [28]. They can have up to 4 broods, so by mid-August, early broods may be 2–3 months old, capable of flight and fully independent.

Individuals were trapped with mist nets and affixed with NTQB-3-2 Avian Nano Tag coded transmitters (0.67 g; 124 day expected tag life; Lotek Wireless Inc., Newmarket, ON) using a leg-loop harness [31]. Sparrows have an average mass of 22.5 g so tag weight did not exceed 3 % of their total body weight [32]. We saw no evidence of altered behaviour or mortality several days after tagging.

Four automated telemetry towers (14 antennas) with SensorGnome receivers (www.sensorgnome.org) were operated on Sable Island during the period of the study. An additional 30 receiver stations were situated on the Atlantic coast between Northern Nova Scotia and Cape Cod, Massachusetts (Fig. 1). Although not all of these towers were operational during the entire study (see Additional file 1 for details on towers) a sufficient number of them were present and operational to detect individuals moving through the northern and southern parts of the Gulf of Maine. Receivers in Nova Scotia towers were situated between 20–50 km apart along the coast in elevated areas with unobstructed views of the ocean.

### Analysis

Data initially recorded by SensorGnomes contain a proportion of false positives in order to reduce the number of missed detections. Data were post-processed following methods described by [16, 33]. We initially included only sets of 3 consecutive detections of a tag that were separated by the specific burst interval for each tag. We then specifically examined some sets of 2 subsequent detections, and included two of these sets where the location of the detection was consistent with the movement of the bird (as determined by the sets of 3 subsequent detections).

Although both adult males and females were tagged we could not make comparisons between the sexes because only 11 adults were detected on the mainland. Furthermore, we did not assess mass or fat content differences between individuals because birds were tagged at least one month prior to departure, and so measurements would not reflect the true measures at the time of departure.

To determine the timing of initiation of migration, we used the date of first detection on mainland Nova Scotia, which assumes that the initiation of migration is in fact the departure of an individual from Sable Island. Most individuals (27/39) were detected on the mainland within 12 h of their final detections on Sable Island, and 37/39 were detected within 100 h of departure. We similarly calculated the date of final detection in Nova Scotia.

We calculated bearings for two types of overwater flights, those from Sable Island to mainland Nova Scotia and those from the southernmost point in mainland Nova Scotia (Bon Portage Island; BPI; Fig. 1) across the Gulf of Maine. We calculated the rhumb-line bearing (package geosphere [34]) between the locations of the respective towers at the ends of the presumed flight path. Bearings were not calculated for birds with final detections at stations to the northeast of BPI because they may have taken overland routes which would not provide true overwater bearings. Bearings were only calculated for individuals that were first detected <100 h after their last detection prior to crossing the water body. Most individuals were detected within 12 h of departing Sable Island; for those that did not, we assume that they reached the respective mainland and initiated a stopover. Two individuals arrived 9 and 18 days after their last detection (longer than the average stopover length) and so were removed because there is considerable uncertainty about what they did in the intervening time. To classify a flight as direct or not, we calculated ground speed (the rhumb-line distance between two consecutive detections at two different sites divided by the time between detections).

When an individual was detected at a receiver to the northeast of a previous detection, we considered it to have made a ‘reverse migration’. When an individual was detected at two adjacent receivers > 10 h apart, or where detections at the same receiver spanned > 8 h with no detections at other receivers, we considered an individual to have ‘stopped over’. Detection range varies considerably depending primarily on an individual’s behaviour; those on the ground are only detected within a range of a few km, whereas those in flight can be routinely detected up to 15 km distant. Distances between adjacent receivers in Nova Scotia were between 9 and 48 km so at the majority of sites an individual would not be detected while stationary at a stopover, but would be detected upon resuming migration. We excluded detections at Cape Cod from consideration for stopover, where most detections spanned >16 days (compared to a maximum of 10 days at all other sites; Table 1). This clear difference in stopover behaviour, and that the location is within the wintering area of Ipswich Sparrows, suggests that these individuals had completed their migration. Thus, they were excluded to ensure stopover analyses did not include overwintering birds that had ceased migrating.

**Table 1:** Mean number and duration of stopovers, and flight distance between stopovers of adult and juvenile Ispwich Sparrows detected on the mainland

	Mean number of stopovers	Mean stopover duration up to Cape Cod (days)	Mean stopover duration at Cape Cod (days)	Mean flight distance (km) between stopovers
Adult	1.6	2.8 (*n* = 17)	4 (*n* = 1)	319.6
Juvenile	3.8	5.8 (*n* = 103)	28 (*n* = 4)	237.0

We fit generalized linear models (glms) to test the hypothesis that the timing of initiation of migration (glm with normal errors) and the number of stopovers (glm with Poisson errors) depended on age. For stopover duration, there were multiple responses for an individual (5/11 adults and 22/28 juveniles had >1 stopover) so we fit generalized linear mixed models (normal errors) with individual as a random effect (package lme4 [35]) to test the hypothesis that stopover duration depended on age. In the results we present the coefficient for the age effect, using ‘adult’ as the reference state.

For analysis of the bearings of individuals crossing water, we used circular analysis of variance (package circular [36]) to test whether the means of the bearings depended on age and Watson’s two-sample test of homogeneity (package CircStats [37]) to test whether the variance of bearings depended on age. For two-by-two contingency tables we used Fisher’s Exact Test for count data.

## Results

Ipswich Sparrows remained on Sable Island for 31–86 days after tagging. On the mainland we detected 28/33 (85 %) of juveniles but only 11/31 (30 %) of adults. All individuals detected on the mainland were initially detected in Nova Scotia (Table 2); 29 of these (4 adults, 25 juveniles) were subsequently detected in coastal areas of the Gulf of Maine.

**Table 2:** Total number of tagged Ipswich Sparrows detected by location on the mainland

	Adults	Juveniles	Total
Total tagged	31	33	64
Detected in Nova Scotia	11	28	39
*Final NS detection on BPI*	7	15	22
*Final NS detection not BPI*	4	13	17
Detected around Gulf of Maine*	4	25	29
*Initial detection in N Maine*	0	12	12
*Initial detection in S Maine*	4	13	17

*All birds detected around the Gulf of Maine were previously detected in Nova Scotia, all birds detected on the mainland were first detected in Nova Scotia

### Timing of the first migratory flight

Juveniles (*n* = 28) were first detected on the mainland between 17 September and 23 October ($\overline{x} = 3$ October). Adults (*n* = 11) were first detected on the mainland between 18 October and 11 November ($\overline{x} = 27$ October), ~24 days after juveniles (Gaussian glm; date of initiation of migration; β (juveniles in relation to adults) = −24.0 ± 3.15, *p* < 0.001). Adults and juveniles remained temporally separate during migration through Nova Scotia (Gaussian glm; date of last detection in mainland Nova Scotia; β (juveniles in relation to adults) = −20.8 ± 2.79, *p* < 0.001).

### Selection of route

**Fig. 2:**
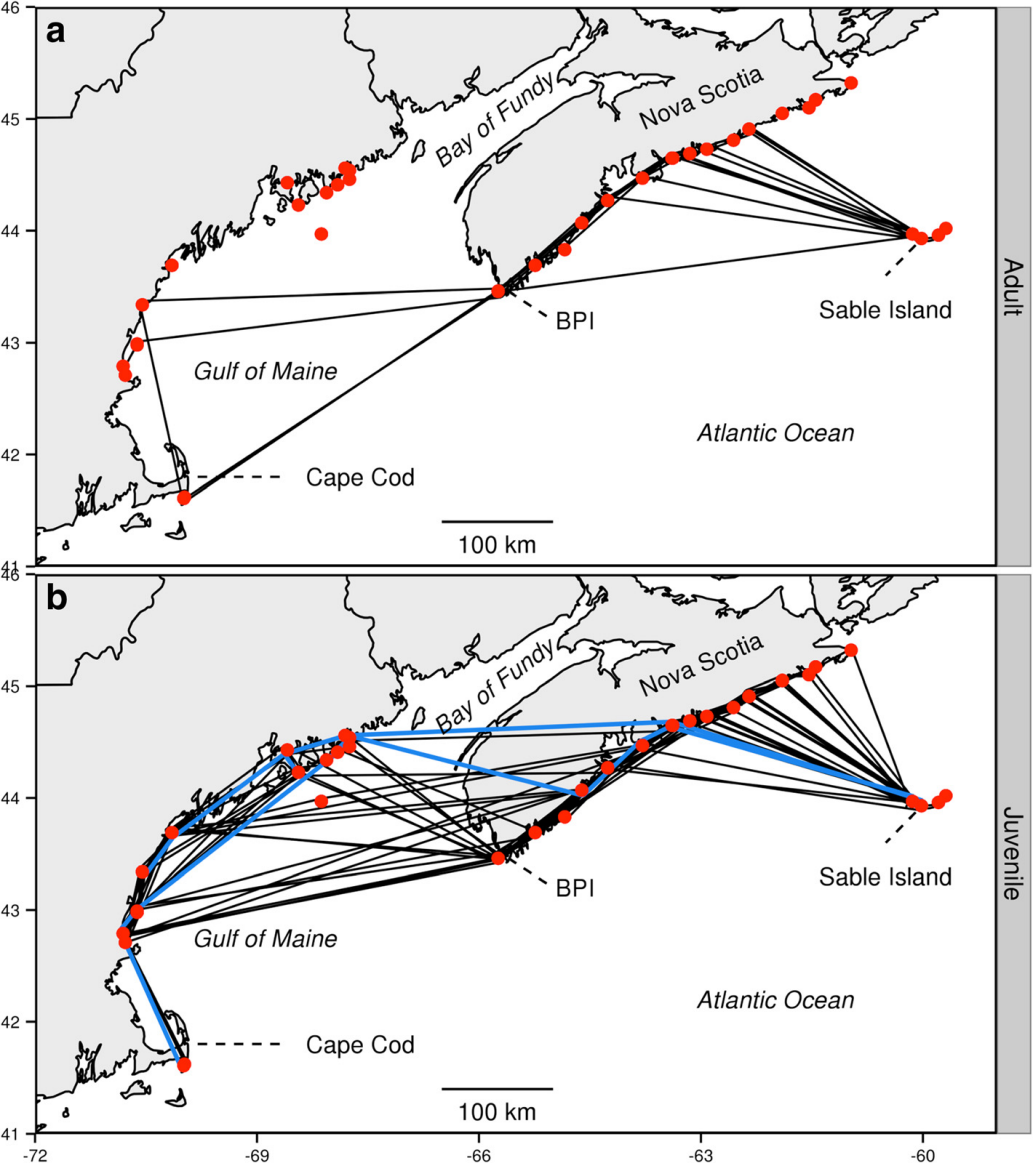
Map of adult and juvenile Ipswich Sparrow migratory routes. Observed routes of (**a**) adult (*n* = 11) and (**b**) juvenile (*n* = 28) Ipswich Sparrows. Direct flights by two juveniles across mainland Nova Scotia indicated by *blue lines*. Receiver stations denoted by *red circles*. Bon Portage Island = BPI

There was evidence of different routes between adults (Fig. 2a) and juveniles (Fig. 2b) based on overwater orientations, and points of detection along the coast of Nova Scotia and along the Gulf of Maine region. All 39 tracked migratory journeys began with an initial overwater flight between Sable Island and mainland Nova Scotia. Most of these (28/39; 72 %) were detected within the first 12 h suggesting that the receiver array was effective in detecting direct overwater flights between Sable Island and initial mainland landfall. The angle of orientation of these initial flights differed slightly between adults and juveniles (288° vs. 299°; circular analysis of variance; *p* = 0.03) but there was no evidence for differences in variability (rho = 0.018 vs. 0.026; Watson’s two-sample test for homogeneity = 0.05, *p* > 0.1).

After reaching mainland Nova Scotia, most individuals were detected travelling southwest along the southern coastline. The adults (*n* = 4, Fig. 2a) that were detected in coastal Gulf of Maine were last detected in Nova Scotia in the extreme southwest site (BPI) suggesting that they flew across the Gulf of Maine. About half (12/25) of the juveniles detected in the Gulf of Maine were last detected in Nova Scotia at BPI, the rest (13/25) were last detected at 5 different sites in coastal Nova Scotia ~50 to 230 km to the northeast of BPI. These individuals likely flew via a partially overland route that circumvented all or part of the Gulf of Maine. Two of these individuals were detected on the northern Gulf of Maine < 8 h after their last detections in Nova Scotia (ground speeds of 10 and 13 m/s). These speeds suggest that the flights were direct, and thus primarily overland across mainland Nova Scotia with a short over-water crossing at the Bay of Fundy (Fig. 2b). The other 10 juveniles detected on the Gulf of Maine were observed 1–21 days after their last detections in Nova Scotia; the lack of detections at other sites along the southwest coast of Nova Scotia suggests that they too made flights crossing mainland Nova Scotia and the Bay of Fundy to reach the upper Gulf of Maine. Of the remaining 10 individuals that were never detected on the Gulf of Maine, BPI was the most frequent last site of detection (3/7 adults, 3/3 juveniles) suggesting possible direct over-water flights terminating south of Cape Cod, the point furthest south in our telemetry array, or unsuccessful flights across open water.

A significantly higher proportion of juveniles than adults were detected on the Gulf of Maine (89 % vs. 36 %; *p* = 0.01, Fisher’s exact test; *p* = 0.002). Although we have no evidence that the proportion of adults and juveniles detected at the northerly Gulf of Maine sites (adults *n* = 0, juveniles *n* = 12) differed from that at the more southerly ones (adults *n* = 4, juveniles *n* = 13; Fisher’s exact test; *p* = 0.12) our power to detect a difference was low. We point out however that none of the four adults detected in the Gulf of Maine were detected at the more northerly receiver stations. Furthermore, adults departing from BPI had a significantly more southerly orientation across the Gulf of Maine than did juveniles (252° vs. 282°; circular analysis of variance; *p* = 0.01). There was no evidence that the variability in bearings differed by age (rho = 0.026 vs. 0.051; Watson’s two sample test for homogeneity = 0. 09, *p* > 0.1).

### Migratory behaviour and stopover

Adults had fewer (1.6 ± 0.36 vs. 3.8 ± 0.36; poisson glm; β = 0.49 ± 0.24, *p* = 0.037) and shorter (2.8 ± 1.03 vs. 5.8 ± 0.71 days; Gaussian glm; β = 2.8 ± 1.11, *p* < 0.03) stopovers than juveniles (Table 1). There were more stopovers at BPI than elsewhere in Nova Scotia (19 vs. 5.3 ± 1.03, poisson glm; β = 1.66 ± 0.33, *p* < 0.001) but their lengths were similar (Gaussian glm; β = 0.03 ± 0.28 days, *p* = 0.92).

All 3 direction reversals were by juveniles. Two occurred towards the end of a southward migratory flight just prior to a stopover event, and the third occurred at the initiation of a southward migratory flight after a 17 day stopover. The distance between the two consecutive detections comprising the direction reversal ranged from 18–34 km.

## Discussion

During fall migration juvenile Ipswich Sparrows departed 24 days prior to adults, had more and longer stopovers, and avoided overwater flights. Adult Ipswich sparrows undertook longer overwater flights than juveniles (both from Sable Island to mainland Nova Scotia and from southwest Nova Scotia across the Gulf of Maine) and some juveniles, but no adults, moved across mainland Nova Scotia to reach the northern Gulf of Maine—crossing or circumnavigating the Bay of Fundy. Collectively, these results suggest that compared to adults, juvenile Ipswich Sparrows spend more time in a migratory state and have fewer and shorter over-water crossings in their fall migrations. One plausible hypothesis that accounts for these differences is that the initial migratory route of Ipswich Sparrows is innate and that as individuals age, these routes change [22].

Age-specific differences in migratory routes have rarely been shown in birds [26, 38, 39] and even more rarely for passerines [40]. Blackpoll warblers from southwest Nova Scotia show dramatically different post-fledging movements that appear to result in differing migratory routes [40]. Juvenile honey buzzards follow a more direct route than adults [26] whereas juvenile sharp-tailed sandpipers take a longer, less direct route [38]. In all cases juveniles are likely following an ancestral route and alternative (learned) routes may have inherent differences in weather conditions, predation, or food availability that make them more attractive [41–43]. Since juvenile Ipswich Sparrows depart nearly a month earlier than adults, it is also possible that different routes arise because of inherent differences in the timing of departure from Sable Island related to different physiological needs. For example, adults may leave later than juveniles because they must moult or because they require additional energy after breeding.

Route choice may also be attributable to different perceptions of risk. Routes with longer overwater flights likely have increased risks associated with orientation, weather, and fuel loads, but lowered risks associated with shorter routes (less time in unfamiliar habitats and less exposure to poor weather, predators and parasites [44, 45]). Although we cannot directly assess the actual risks of the routes selected by individuals, one hypothesis that would support the observation that juveniles take routes that reduce or eliminate water crossings is that there has been selection to reduce navigational risk in the first migratory journey and(or) to reduce time spent migrating in the second and subsequent journeys [46]. Observations of foraging behaviour and predator presence at stopover, and finer-scale data on actual routes selected will ultimately be required to tease apart the relative importance of these factors in determining route selection.

Both adults and juveniles were more likely to stopover when they encountered the Gulf of Maine which may suggest a behavioural response to the presence of the Gulf of Maine. If so, the fact the lengths of stopovers at the Gulf of Maine did not vary from the length at other locations, suggests that the amount of fat required for that crossing is not greater than that required for other migratory flights. Thus, if there is a perceived risk to the Gulf of Maine, it is likely related to navigational errors, and not to fuel load. Individuals are known to reduce the risks of crossing large water bodies by remaining at, or returning to, the mainland and subsequently initiating water crossings early in the night, with better body condition, or under better weather conditions [23, 25].

Juveniles had significantly more, and longer stopover events than adults indicating that stopover behaviour may also relate to energy requirements, abilities, and experience that differ with age. Individuals arriving at stopovers in lower energetic condition may stay longer in order to accumulate adequate energy reserves [23]. Furthermore, only juveniles were observed moving in directions along the coast opposite to that expected for the season. Two of these reverse flights occurred immediately before a stopover event, suggesting that these individuals were searching for a stopover site with better foraging opportunities, less competition or fewer predators [47]. The third reversal occurred after a 17 days stopover which is more consistent with landscape-scale stopover movements that have been observed in other species [16, 48]. The lack of such movements by adults suggests an awareness of suitable stopover locations and/or comparatively better body condition than juveniles [49, 50].

We detected the majority of juveniles but many fewer adults leaving Sable Island. The possible reasons for the discrepancy include differential tag loss, post-breeding mortality, or direct adult migratory movements beyond the southern edge of the telemetry array. Demographic studies of passerines generally show much higher rates of yearly mortality for juveniles than adults [51], but it is difficult to determine mortality during post-fledging and migration in small passerines. It is possible that adult Ipswich sparrows suffer increased mortality immediately post-breeding [52]. Unlike many passerines at these latitudes, Ipswich sparrows may have as many as 4 broods in a season [28]. Such high reproductive output may directly cause increased mortality or indirectly exacerbate ‘tag effects’ also resulting in increased mortality. Alternatively, weight losses associated with breeding and moulting could increase the likelihood that adults lose their tags relative to juveniles.

We suspect that adults present in coastal Nova Scotia but not detected in the Gulf of Maine flew directly to make landfall at points farther south than the southern extent of the telemetry array, and so were never again detected. It follows therefore that some adults may have flown directly from Sable Island to more southern wintering grounds than our array could detect. Continued study with increased receiver coverage farther south will aid in determining the fate of this large portion of missing adults.

## Conclusion

Due to difficulties in tracking small songbirds, differences in routes between age classes have not been previously shown. Our results suggest that juvenile Ipswich Sparrows may initially select routes based on innate knowledge and may alter routes in following years based on experience or social learning [22]. While differences in routes and stopover behaviour may be attributable to differences in experience, condition, and temporal difference, both adults and juveniles are more likely to stopover at water barriers than at other sites, suggesting over-water crossings are perceived as risky to both age classes.

## Availability of supporting data

The data set supporting the results of this article is available in the Motus repository, [http://www.motus-wts.org].

## Additional file

Additional file 1:
**Receiver station locations and antenna orientations.** Start and Stop times refer to the period each receiver was active. Some changes in antenna orientation occurred (eg. CANS1 & CANS2) however sites remained active during orientation changes and therefore were unlikely to impact detections during this study. Receivers were maintained and checked for operation on a cycle of weeks or months depending on site accessibility. Evaluation of raw data showed no major receiver malfunctions except at 3 locations, all of which should not have affected data: CANS1 was non-operational for 3 days (Nov 10–13) however CANS2 was within 1 km and was operational during this time; KEJI was non-operational for 8 days (between Nov 8–19) however only one individual was detected for the first time after Nov 8th and it was first detected 150 km NE at CONRD, thus this likely did not affect overwater flight information from Sable Island to the mainland; STAGE had frequent blackouts however LOT1 was within 5 km. (XLS 37 kb)

## Abbreviations

BPI: Bon Portage Island
